# Generation of Marker-Free Transgenic Rice Resistant to Rice Blast Disease Using *Ac/Ds* Transposon-Mediated Transgene Reintegration System

**DOI:** 10.3389/fpls.2021.644437

**Published:** 2021-04-20

**Authors:** Xin Li, Longyu Pan, Dongling Bi, Xudan Tian, Lihua Li, Zhaomeng Xu, Lanlan Wang, Xiaowei Zou, Xiaoqing Gao, Haihe Yang, Haiyan Qu, Xiangqian Zhao, Zhengjie Yuan, Haiyan He, Shaohong Qu

**Affiliations:** ^1^State Key Laboratory for Managing Biotic and Chemical Threats to the Quality and Safety of Agro-Products, Institute of Virology and Biotechnology, Zhejiang Academy of Agricultural Sciences, Hangzhou, China; ^2^College of Chemistry and Life Sciences, Zhejiang Normal University, Jinhua, China; ^3^College of Plant Protection, Hunan Agricultural University, Changsha, China; ^4^Institute of Crop Science and Nuclear Technology Utilization, Zhejiang Academy of Agricultural Sciences, Hangzhou, China

**Keywords:** rice, rice blast (*Magnaporthe oryzae*), disease resistance gene, selection marker, *Ac/Ds* transposable element, marker-free transgenic plant

## Abstract

Rice blast is one of the most serious diseases of rice and a major threat to rice production. Breeding disease-resistant rice is one of the most economical, safe, and effective measures for the control of rice blast. As a complement to traditional crop breeding, the transgenic method can avoid the time-consuming process of crosses and multi-generation selection. In this study, maize (*Zea mays*) Activator (*Ac*)/Dissociation (*Ds*) transposon vectors carrying green fluorescent protein (GFP) and red fluorescent protein (mCherry) genetic markers were used for generating marker-free transgenic rice. Double fluorescent protein-aided counterselection against the presence of T-DNA was performed together with polymerase chain reaction (PCR)-based positive selection for the gene of interest (GOI) to screen marker-free progeny. We cloned an RNAi expression cassette of the rice *Pi21* gene that negatively regulates resistance to rice blast as a GOI into the *Ds* element in the *Ac/Ds* vector and obtained marker-free T1 rice plants from 13 independent transgenic lines. Marker-free and *Ds*/GOI-homozygous rice lines were verified by PCR and Southern hybridization analysis to be completely free of transgenic markers and T-DNA sequences. qRT-PCR analysis and rice blast disease inoculation confirmed that the marker-free transgenic rice lines exhibited decreased *Pi21* expression levels and increased resistance to rice blast. TAIL-PCR results showed that the *Ds* (*Pi21*-RNAi) transgenes in two rice lines were reintegrated in intergenic regions in the rice genome. The *Ac/Ds* vector with dual fluorescent protein markers offers more reliable screening of marker-free transgenic progeny and can be utilized in the transgenic breeding of rice disease resistance and other agronomic traits.

## Introduction

Rice blast caused by the rice blast fungus (*Magnaporthe oryzae*) is one of the most serious diseases of rice and a major threat to rice production (Deng et al., 2017). Breeding disease-resistant rice is one of the most economical, safe, and effective measures for the control of rice blast. More than 100 genes or loci conferring rice blast resistance have been identified (Li W. T. et al., 2019), of which 31 resistance genes have been cloned (Xiao et al., 2020). Most of the cloned rice blast resistance genes are classic dominant R genes. Although R genes exhibit a phenotype of high resistance or immunity to rice blast, they usually confer resistance to a narrow spectrum of rice blast races and their resistance is easily lost due to the variation in pathogenicity of *M. oryzae*. The *Pi21* gene negatively regulates rice blast resistance, and rice cultivars carrying a recessive *pi21* gene show partial resistance (incomplete resistance) to *M. oryzae* (Fukuoka et al., 2009). Although the *pi21* gene does not respond much strongly and rapidly to *M. oryzae* infection as compared with the race-specific R genes such as *Pi9* (Qu et al., 2006) and *Pigm* (Deng et al., 2017), the *pi21*-mediated slow defense response might lead to the durability of disease resistance and can be utilized in rice breeding.

As a complement to traditional crop breeding, the transgenic method can avoid the time-consuming process of crosses and multi-generation selection. Transforming cloned disease resistance genes into susceptible plants can improve disease resistance in a short period. Therefore, the transgenic approach by modifying the expression of the *Pi21* gene is useful for breeding durable disease resistance in rice.

Selection marker genes, such as antibiotic or herbicide resistance genes, are used in plant transformation to screen transformed cells (Sundar and Sakthivel, 2008; Tuteja et al., 2012). However, marker genes should be eliminated after transformation because their presence in transgenic plants may arouse biosafety concerns such as potential harms to human health and to the environment (Tuteja et al., 2012). Developing marker-free transgenic plants can avoid such risks and facilitate the commercialization of transgenic crop plants.

Methods of removing selection markers from transgenic plants include co-transformation, site-specific recombination, and transposition (Tuteja et al., 2012). In co-transformation, T-DNA containing a selection marker gene and a T-DNA carrying gene of interest (GOI) are simultaneously transformed into plants. To eliminate the marker gene, transgenic progeny in which the GOI T-DNA has segregated from the marker-gene T-DNA are selected (Ling et al., 2016; Pan et al., 2020). In the Cre/lox strategy (Bai et al., 2008; Nandy et al., 2015; Ow, 2016; Zhu et al., 2018; Kleidon et al., 2020), a marker gene flanked by lox sites was deleted after transformation via site-specific recombination, and the GOI was retained by transgenic plants. Similarly, in another study a selection marker and a CRISPR/Cas9 expression cassette were placed between two gRNA target sites in a T-DNA sequence and deleted via double breakage at the target sites (Wang et al., 2019). Additionally, the maize (*Zea mays*) Activator/Dissociation (*Ac/Ds*) transposon elements (Lazarow et al., 2013) were used to mediate transgene reintegration in transgenic plants (Gao et al., 2015). *Ds*-carrying GOI, *Ac* transposase (*Ac*TPase), and selection marker gene were simultaneously introduced into plants by T-DNA transformation. Under the action of *Ac*TPase, *Ds* is excised from T-DNA and reintegrated at another genomic site. Marker-free transgenic plants that are T-DNA free and carry stabilized *Ds* insertions can be generated after genetic recombination between the T-DNA integration site and the *Ds* reintegration site.

The GOI transposes together with *Ds*, which results in a clear boundary between the reintegrated *Ds* and the plant genome and leaves the GOI sequence intact and unaffected by T-DNA structural variation (Gao et al., 2015). Moreover, transposition allows the GOI to be reintegrated with *Ds* at numerous new insertion sites in transgenic plants. Thus, only a small number of primary transformants are needed to obtain a population of transgenic progeny with independently integrated GOIs, which can then be used to screen for suitable transgene integration sites and to avoid transgene expression levels being affected by positional effects (Matzke and Matzke, 1998).

Gao et al. (2015) introduced green fluorescent protein (GFP) to the *Ac/Ds* transposon vector that improved the efficiency of progeny screening through green fluorescence assay, and developed marker-free rice lines with stable insect-resistance and agronomic traits. However, in practice, some non-fluorescent transgenic progeny derived from the single fluorescent protein-expressing *Ac/Ds* transposon vector (Gao et al., 2015) may still carry transformation marker or T-DNA sequences, due to GFP inactivation by *Ds* transposition into the GFP gene or by GFP sequence rearrangement during T-DNA integration. In this paper, we also explore ways to overcome this disadvantage.

We transformed a *Pi21*-RNAi expression cassette conferring rice blast resistance into a rice cultivar via *Ac/Ds* transposon-mediated transgene reintegration and obtained marker-free transgenic rice resistant to rice blast. We developed a double fluorescent protein-expressing *Ac/Ds* transposon vector carrying GFP and red fluorescent protein (mCherry), improving the reliability of the selection system of transgenic segregating populations and reducing the frequency of generating false marker-free transgenic plants.

## Results

### Construction of a Double Fluorescent Protein-Expressing *Ac/Ds* Vector and Generation of Transgenic Rice Plants

**FIGURE 1:**
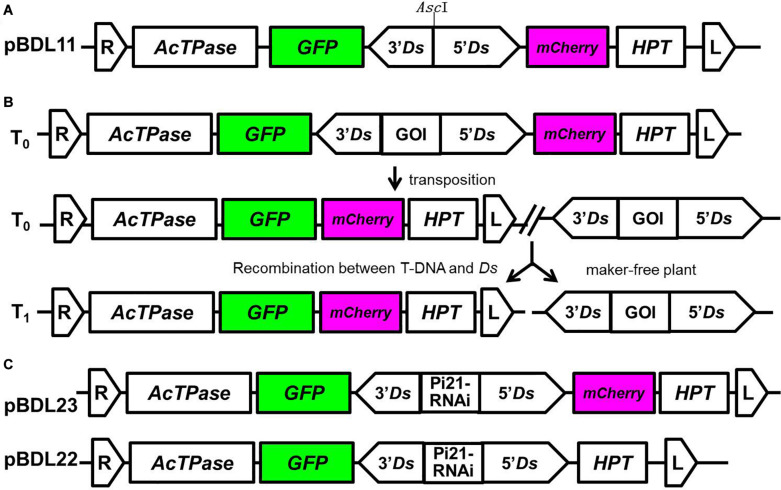
Double-fluorescent protein-expressing *Ac/Ds* transposon vectors for the generation of marker-free transgenic plants. **(A)** pBDL11 is a double-fluorescent protein-expressing *Ac*/*Ds* universal vector for transgene reintegration. pLJ26 (Gao et al., 2015) is a single-fluorescent protein-expressing *Ac*/*Ds* universal vector used to compare the efficacy of screening transgenic progeny with pBDL11. The *Asc*I restriction sites in pBDL11 and pLJ26 were used for insertion of the gene of interest (GOI). **(B)** A diagram of *Ds* transposition-mediated transgene reintegration. The working pattern of the double fluorescent protein-expressing *Ac/Ds* vector is as follows: (i) T-DNA is integrated into the plant genome during transformation; (ii) by the action of *Ac*TPase, *Ds* is excised from T-DNA and reintegrated at another site in the plant genome; (iii) T1 progeny carrying reintegrated *Ds* elements, from which T-DNA sequences including HPT and *Ac*TPase are removed, are generated by genetic recombination. **(C)** The *Ac*/*Ds* vectors pBDL23 and pBDL22, in which *Ds* elements harbor an RNAi cassette of the rice gene *Pi21* conferring rice blast disease resistance, were constructed to generate marker-free disease-resistant transgenic rice plants.

We constructed a double fluorescent protein-expressing *Ac/Ds* transposon vector in which the *Ds* element carrying a GOI is tandemly linked to *Ac* transposase (*Ac*TPase), hygromycin phosphotransferase (HPT), GFP, and mCherry (pBDL11; Figure 1A). Compared with a single fluorescent protein-expressing *Ac/Ds* transposon vector (pLJ26; Gao et al., 2015), pBDL11 carries an mCherry fluorescent protein-expression cassette in addition to the GFP cassette.

The working pattern of the double fluorescent protein-expressing *Ac/Ds* vector is shown in Figure 1B. T-DNA-harboring T1 progeny can be identified by GFP and mCherry fluorescence assays, and non-fluorescent T1 progeny can be screened by PCR assays for *Ds* and the GOI to obtain marker-free transgenic plants.

In the *Ds* element, a *Pi21*-RNAi expression cassette that confers resistance to rice blast disease (*Magnaporthe oryzae*) was inserted into the *Asc*I site as a GOI to construct pBDL23 (Figure 1C). *pi21* is a recessive rice gene conferring broad spectrum and durable resistance to rice blast. RNAi knockdown of the dominant *Pi21* gene was shown to confer rice blast resistance (Fukuoka et al., 2009; Zhang et al., 2016). For comparison and parallel control experiments, the *Pi21*-RNAi cassette was cloned into the single fluorescent protein-expressing *Ac/Ds* vector pLJ26 (Gao et al., 2015) to construct pBDL22 (Figure 1C).

**FIGURE 2:**
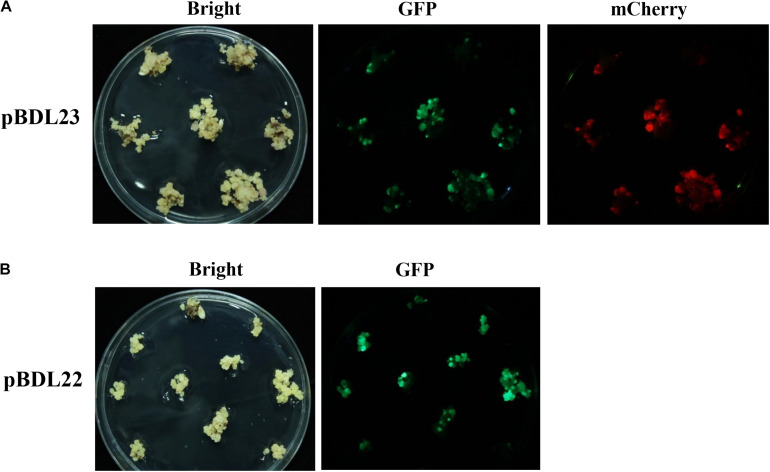
Rice calli transformed with fluorescent protein-expressing *Ac/Ds* transposon vectors. **(A)** Rice calli were co-cultivated with *Agrobacterium* carrying the double-fluorescent protein-expressing Ac/Ds transposon vector pBDL23, and then were selected on hygromycin medium for 19 days. Hygromycin-resistant (Hyg+) calli were picked up for a second-round selection for 20 days and assayed for GFP and mCherry fluorescence. **(B)** pBDL22 derived from the single-fluorescent protein-expressing *Ac/Ds* transposon vector pLJ26 (Gao et al., 2015) was transformed into rice in parallel control experiments.

pBDL23 and pBDL22 were transformed into rice cultivar Nipponbare via *Agrobacterium*. Rice calli were co-cultivated with *Agrobacterium* strains for 3 days and then cultured on hygromycin medium for 19 days for first-round selection. The rice calli expressing GFP and mCherry fluorescence were counted. The transformation efficiencies for pBDL23 and pBDL22 were 67.26% and 59.78%, respectively (Table 1). The hygromycin-resistant calli were subjected to a second-round selection culture for 20 days (Figure 2). The pBDL23-transformed calli expressing GFP and mCherry fluorescence and the pBDL22 calli expressing GFP were selected and cultured on plant regeneration medium to obtain rice primary transformants (T0 plants) with regeneration rates of 55.17% and 48.58%, respectively (Table 1). Subsequently, healthy double-fluorescent pBDL23-transformed plants and GFP-fluorescent pBDL22 plants were transplanted to soil until maturity, after which T1 rice seeds could be harvested.

**TABLE 1:** Transformation and differentiation rates of pBDL23- and pBDL22-transformed rice calli.

Vector	Initial calli	Transformed calli *	Transformation rates (%)	Differentiated calli **	Calli differentiated into plants	Differentiation rates (%)	Plants transplanted to soil ***
pBDL23	562	378	67.26	232	128	55.17	73
pBDL22	557	333	59.78	212	103	48.58	79

*Nipponbare rice calli were co-cultured with *Agrobacterium* for 3 days and subjected to 19 days of selection culture on hygromycin medium. The pBDL23-transformed calli expressing GFP and mCherry fluorescence and the pBDL22-transformed calli expressing GFP fluorescence were counted separately. For each vector, the transformation rate is the percentage of the transformed fluorescent calli among the calli initially cultured on hygromycin selection medium. **Transformed fluorescent calli described as above were subjected to a second selection culture for 20 days, and then cultured on differentiation medium for 48 days to be regenerated into plants. For each vector, the differentiation rate is the percentage of the fluorescent calli that were regenerated into plants among the fluorescent calli initially cultured on differentiation medium. ***Healthy pBDL23-transformed plants expressing GFP and mCherry and pBDL22-transformed plants expressing GFP were transplanted into soil, and T1 seeds were harvested at maturity.

**FIGURE 3:**
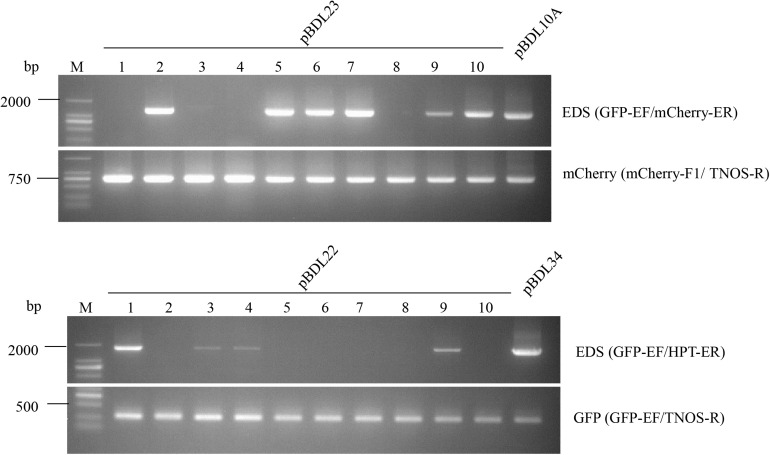
PCR of the *Ds* empty donor site (EDS), mCherry, and GFP sequences in T0 rice plants transformed with pBDL23 and pBDL22. Ten pBDL23- or pBDL22-transformed T0 rice plants (1–10) were tested. *Ds* excision in T0 plants was monitored by the EDS bands. pBDL10A and pBDL34 are the progenitor vectors of pBDL23 and pBDL22 before a *Ds* element was inserted into the *Sac*I restriction site (see the section “Materials and Methods”), and were used as controls in EDS-PCR for the pBDL23- and pBDL22-transformed plants, respectively. GFP-EF and mCherry-ER are primers specific to the sequences adjacent to *Ds* element in pBDL23. GFP-EF and HPT-ER are primers specific to the sequences adjacent to *Ds* element in pBDL22.

To test the transposition activity of the *Ds* elements, transposon empty donor sites (EDS) in pBDL23- and pBDL22-transformed rice plants were amplified using specific PCR primers (Figure 3). EDS sequences were amplified in 60% and 40% of the pBDL23- and pBDL22-transformed T0 plants, respectively, suggesting the transposability of the *Ds* elements in rice.

### Genetic Screening of Marker-Free T1 Transgenic Rice Progeny

T1 seeds of pBDL23-transformed rice lines were germinated and assayed for mCherry and GFP fluorescence. GFP and mCherry segregation ratios of the T1 lines were calculated, and chi-square fitting analyses for Mendelian genetic ratios (3:1, 15:1 and 63:1) were carried out for each rice line (Table 2). The T1 seedlings of pBDL22 rice lines were assayed for GFP fluorescence, and the GFP segregation ratios of T1 lines were then analyzed (Table 3). Among 42 pBDL23-transformed rice lines, 31 (73.8%) lines from the T1 generation showed Mendelian segregation ratios of 3:1, 15:1, or 63:1 for both GFP and mCherry; in five rice lines, neither GFP nor mCherry segregated with Mendelian ratios (Table 2). Five pBDL23-transformed rice lines showed Mendelian segregation ratios for GFP but not for mCherry, and one pBDL23 rice line showed Mendelian ratios for mCherry but not for GFP. Of 35 pBDL22-transformed rice lines, 32 (91.4%) showed GFP segregation with Mendelian ratios in the T1 generation (Table 3).

**TABLE 2:** Fluorescence assays and PCR analysis of T1 transgenic rice plants transformed with pBDL23.

Line no.	GFP and mCherry fluorescence assays										PCR analysis of non-fluorescent plants						
	G^–^R^–^	G^–^R^+^	G^+^R^–^	G^+^R^+^	*GFP*: χ^2^(3:1)	*GFP*: χ^2^(15:1)	*GFP*: χ^2^(63:1)	*mCherry*: χ^2^(3:1)	*mCherry*: χ^2^(15:1)	*mCherry*: χ^2^(63:1)	Plants	*Pi21* RNAi^+^	*GFP*^+^	*HPT*^+^	*AcTPase*^+^	MF	false MF
14YD262-1	0	0	14	29	13.03b	1.90	0.04	0.94	46.40b	248.82b	0	ND	ND	ND	ND	0	0
14YD263-1	29	1	0	58	3.41	111.71b	584.42b	2.56	102.59b	543.60b	1	0	0	0	0	1	0
14YD264-2	24	0	0	71	0.00	55.41b	331.71b	0.00	55.41b	331.71b	1	0	0	0	0	1	0
14YD265-1	23	0	1	75	0.08	45.87b	288.32b	0.00	51.67b	316.50b	0	ND	ND	ND	ND	0	0
14YD266-3	7	0	0	23	0.00	12.17b	78.83b	0.00	12.17b	78.83b	0	ND	ND	ND	ND	0	0
14YD268-1	6	0	15	60	12.45b	0.04	14.39b	0.00	50.21b	296.95b	0	ND	ND	ND	ND	0	0
14YD269-1	25	0	0	67	0.13	65.22b	375.88b	0.13	65.22b	375.88b	0	ND	ND	ND	ND	0	0
14YD270-1	25	0	0	51	2.12	87.59b	464.93b	2.12	87.59b	464.93b	13	0	0	0	0	13	0
14YD271-3	32	0	0	59	4.49a	124.96b	646.37b	4.49a	124.96b	646.37b	8	0	0	0	0	8	0
14YD272-1	21	0	61	0	0.00	49.20b	292.86b	242.02b	1214.05b	5102.20b	0	ND	ND	ND	ND	0	0
14YD273-2	29	0	0	35	13.02b	160.07b	768.25b	13.02b	160.07b	768.25b	14	0	0	0	0	14	0
14YD275-1	24	0	0	71	0.00	55.41b	331.71b	0.00	55.41b	331.71b	0	ND	ND	ND	ND	0	0
14YD276-1	19	0	0	60	0.00	39.74b	245.33b	0.00	39.74b	245.33b	7	0	0	0	0	7	0
14YD278-1	25	0	0	61	0.56	72.59b	405.38b	0.56	72.59b	405.38b	0	ND	ND	ND	ND	0	0
14YD279-3	26	0	0	68	0.23	69.93b	399.43b	0.23	69.93b	399.43b	0	ND	ND	ND	ND	0	0
14YD280-2	6	9	17	66	4.41a	12.21b	111.58b	0.05	46.70b	291.70b	0	ND	ND	ND	ND	0	0
14YD289-1	22	0	0	43	2.26	79.84b	419.71b	2.26	79.84b	419.71b	0	ND	ND	ND	ND	0	0
14YD294-1	25	0	0	48	2.85	92.93b	485.98b	2.85	92.93b	485.98b	0	ND	ND	ND	ND	0	0
14YD303-1	19	0	0	48	0.24	52.18b	295.59b	0.24	52.18b	295.59b	0	ND	ND	ND	ND	0	0
14YD305-2	27	0	0	66	0.61	78.54b	438.57b	0.61	78.54b	438.57b	0	ND	ND	ND	ND	0	0
14YD306-1	13	0	0	50	0.43	19.86b	136.85b	0.43	19.86b	136.85b	1	0	0	0	0	1	0
14YD307-1	14	0	0	21	3.44	62.40b	311.67b	3.44	62.40b	311.67b	0	ND	ND	ND	ND	0	0
14YD309-1	5	0	73	0	13.40b	0.03	8.97b	230.02b	1154.05b	4850.21b	0	ND	ND	ND	ND	0	0
14YD310-1	18	0	0	58	0.02	36.51b	227.64b	0.02	36.51b	227.64b	3	0	0	0	0	3	0
14YD311-1	3	9	9	39	0.56	17.08b	120.89b	0.56	17.08b	120.89b	0	ND	ND	ND	ND	0	0
14YD312-1	18	0	42	33	1.29	25.07b	180.02b	75.36b	528.95b	2355.56b	0	ND	ND	ND	ND	0	0
14YD313-1	23	0	0	48	1.69	78.42b	418.99b	1.69	78.42b	418.99b	0	ND	ND	ND	ND	0	0
14YD315-1	25	0	0	38	6.48a	114.54b	570.68b	6.48a	114.54b	570.68b	1	0	0	0	0	1	0
14YD316-1	25	0	0	62	0.46	71.28b	400.18b	0.46	71.28b	400.18b	0	ND	ND	ND	ND	0	0
14YD318-1	19	0	70	1	0.53	31.43b	211.08b	258.13b	1302.43b	5479.63b	0	ND	ND	ND	ND	0	0
14YD319-1	19	0	0	63	0.07	37.23b	235.08b	0.07	37.23b	235.08b	0	ND	ND	ND	ND	0	0
14YD320-1	6	13	0	70	0.45	32.10b	213.84b	14.87b	0.00	12.34b	0	ND	ND	ND	ND	0	0
14YD321-1	29	0	0	38	10.99b	150.57b	731.35b	10.99b	150.57b	731.35b	0	ND	ND	ND	ND	0	0
14YD323-1	20	0	0	54	0.07	51.03b	295.64b	0.07	51.03b	295.64b	0	ND	ND	ND	ND	0	0
14YD325-2	5	14	15	59	0.81	29.54b	203.15b	0.43	34.38b	227.69b	0	ND	ND	ND	ND	0	0
14YD326-2	20	0	0	75	0.59	33.04b	222.12b	0.59	33.04b	222.12b	0	ND	ND	ND	ND	0	0
14YD328-1	11	0	0	36	0.01	20.77b	131.92b	0.01	20.77b	131.92b	0	ND	ND	ND	ND	0	0
14YD330-1	4	0	0	68	13.50b	0.00	5.09a	13.50b	0.00	5.09a	1	0	0	0	0	1	0
14YD335-1	27	0	0	53	2.82	98.61b	518.15b	2.82	98.61b	518.15b	16	0	0	0	0	16	0
14YD343-1	25	0	52	3	1.35	81.12b	439.31b	212.82b	1090.61b	4601.96b	2	0	0	0	0	2	0
14YD344-2	32	0	50	0	7.87b	144.78b	724.03b	242.02b	1214.05b	5102.20b	0	ND	ND	ND	ND	0	0
14YD347-1	16	0	0	50	0.00	33.46b	206.22b	0.00	33.46b	206.22b	11	0	0	0	0	11	0

*The ratio of the number of GFP- or mCherry-fluorescent T1 segregants to the number of non-fluorescent siblings in each T1 line was analyzed by a chi-squared test. Mendelian segregation patterns (3:1, 15:1, or 63:1) were accepted at the 0.05 and 0.01 probability levels when the calculated chi-square values are less than the theoretical values of 3.84 and 6.64, respectively. PCR primers for amplification of *Pi21*-RNAi, *GFP*, *mCherry*, *HPT*, and *AcTPase* were listed in Supplementary Table 1. G, *GFP*; R, *mCherry*; +, positive; −, negative; MF, marker-free *Ds*(Pi21-RNAi) plants; false MF, non-fluorescent *Ds*(Pi21-RNAi) plants containing residual T-DNA sequences; a, significant level (0.01 < *P* < 0.05); b, most significant level (*P* < 0.01); ND, not determined.*

**TABLE 3:** Fluorescence assays and PCR analysis of T1 transgenic rice plants transformed with pBDL22.

Line No.	GFP fluorescence assays					PCR analysis of non-fluorescent plants						
	G^+^	G^–^	*GFP*: χ ^2^(3:1)	*GFP*: χ ^2^(15:1)	*GFP*: χ ^2^(63:1)	Plants	*Pi21* RNAi^+^	*GFP*^+^	*HPT*^+^	*AcTPase*^+^	MF	false MF
14YD181-1	87	5	17.75*b*	0.01	6.63*a*	5	0	ND	ND	ND	0	0
14YD182-2	92	5	19.33*b*	0.06	5.97*a*	5	0	ND	ND	ND	0	0
14YD183-2	85	3	20.74*b*	0.78	0.94	3	0	ND	ND	ND	0	0
14YD184-2	82	4	17.92*b*	0.15	3.51	4	0	ND	ND	ND	0	0
14YD186-1	93	2	25.35*b*	2.12	0.00	2	0	ND	ND	ND	0	0
14YD187-2	82	4	17.92*b*	0.15	3.51	4	0	ND	ND	ND	0	0
14YD188-2	98	0	31.35*b*	5.51*a*	0.71	0	0	0	0	0	0	0
14YD189-2	74	22	0.13	42.71*b*	270.90*b*	22	0	ND	ND	ND	0	0
14YD190-1	71	27	0.22	72.30*b*	413.61*b*	27	0	ND	ND	ND	0	0
14YD191-1	64	26	0.53	74.91*b*	419.36*b*	26	0	ND	ND	ND	0	0
14YD192-1	89	0	28.35*b*	4.91*a*	0.58	0	0	0	0	0	0	0
14YD193-1	76	6	12.75*b*	0.03	14.11*b*	6	0	ND	ND	ND	0	0
14YD196-3	55	34	7.58*b*	149.67*b*	753.17*b*	34	0	ND	ND	ND	0	0
14YD197-1	54	30	4.59*a*	119.48*b*	614.97*b*	30	0	ND	ND	ND	0	0
14YD198-1	70	7	9.56*b*	0.63	23.69*b*	7	0	ND	ND	ND	0	0
14YD199-1	69	28	0.58	80.86*b*	452.56*b*	28	12	0	0	0	12	0
14YD201-2	97	2	26.67*b*	2.34	0.00	2	0	ND	ND	ND	0	0
14YD203-1	92	7	16.03*b*	0.02	16.11*b*	7	1	1	0	0	0	1
14YD204-1	68	25	0.09	64.09*b*	371.33*b*	25	0	ND	ND	ND	0	0
14YD206-1	95	0	30.35*b*	5.31*a*	0.66	0	0	0	0	0	0	0
14YD206-1	0	95	281.01*b*	1409.04*b*	5921.17*b*	95	0	ND	ND	ND	0	0
14YD207-1	91	7	15.73*b*	0.02	16.38*b*	7	1	0	0	0	1	0
14YD209-2	85	6	15.48*b*	0.01	11.88*b*	6	2	ND	ND	ND	0	0
14YD210-2	72	26	0.05	65.37*b*	381.14*b*	26	0	ND	ND	ND	0	0
14YD211-1	67	20	0.10	38.79*b*	245.93*b*	20	2	0	0	0	1	0
14YD214-1	49	16	0.01	34.35*b*	209.85*b*	16	0	ND	ND	ND	0	0
14YD216-1	92	2	25.02*b*	2.07	0.00	2	0	ND	ND	ND	0	0
14YD218-1	69	25	0.06	62.98*b*	366.88*b*	25	0	ND	ND	ND	0	0
14YD219-1	70	27	0.28	73.49*b*	418.39*b*	27	16	0	0	0	16	0
14YD221-1	88	1	25.80*b*	3.16	0.01	1	1	0	0	1	0	1
14YD223-1	85	5	17.13*b*	0.00	6.91*b*	5	0	ND	ND	ND	0	0
14YD225-2	92	0	29.35*b*	5.11*a*	0.62	0	0	0	0	0	0	0
14YD228-1	73	18	1.06	26.17*b*	184.69*b*	18	0	ND	ND	ND	0	0
14YD261-1	30	1	6.72*b*	0.11	0.00	1	1	0	0	0	1	0
14YD282-2	39	16	0.30	45.15*b*	253.38*b*	16	8	0	0	0	8	0

*The ratio of the number of GFP-fluorescent T1 segregants to the number of non-fluorescent siblings in each T1 line was analyzed by a chi-squared test. Mendelian segregation patterns (3:1, 15:1, or 63:1) were accepted at the 0.05 and 0.01 probability levels when the calculated chi-square values are less than the theoretical values of 3.84 and 6.64, respectively. PCR primers for amplification of *Pi21-*RNAi, *GFP*, *HPT*, and *AcTPase* were listed in Supplementary Table 1. G, *GFP*; +, positive; *−*, negative; MF, marker free *Ds*(*Pi21*-RNAi) plants; false MF, non-fluorescent *Ds*(*Pi21*-RNAi) plants containing residual T-DNA sequences; a, significant level (0.01 < *P* < 0.05); b, most significant level (*P* < 0.01); ND, not determined.*

For each pBDL23-transformed rice line segregating in the T1 generation, double-fluorescent (GFP+ and mCherry+) and single-fluorescent (GFP+ or mCherry+) plants were discarded. Non-fluorescent T1 plants were assayed by PCR for *Ds* and the GOI (i.e., the *Pi21*-RNAi expression cassette). For *Ds*- and GOI-positive plants, PCR assays of the T-DNA-based GFP, mCherry, HPT, and *Ac*TPase sequences were performed to confirm their absence. To streamline the screening of T1 rice populations, we used DNA pools of five non-fluorescent *Ds*/GOI plants from the same T1 line for extracts of rice genomic DNA (see Materials and Methods for further details). Each DNA pool was subjected to PCR assays for *Ds* and the GOI (Supplementary Image 1A), and further PCR assays confirmed the absence of GFP, mCherry, HPT, and *Ac*TPase (Supplementary Image 1B). After identifying *Ds*-positive pools, individual plants from each DNA pool were analyzed by PCR to screen marker-free transgenic plants—i.e., *Ds*/GOI-positive and T-DNA-absent T1 plants (Supplementary Image 1C for pBDL23). For parallel experiments using the single fluorescent protein-expressing *Ac/Ds* vector pBDL22, single-fluorescent (GFP+) plants were discarded for each T1 rice line, and non-fluorescent plants were assayed by PCR to screen for marker-free *Ds*/GOI plants (Supplementary Image 1D) using the method described in Gao et al. (2015).

A total of 42 pBDL23-transformed T1 rice lines showed detectable mCherry and GFP fluorescence; after PCR screening of *Ds*/GOI, GFP, mCherry, HPT, and *Ac*TPase sequences, marker-free *Ds*/GOI plants were obtained from 13 rice lines (Table 2). Non-fluorescent progeny from the abovementioned 13 T1 lines showed negative PCR results for GFP, mCherry, HPT, and *Ac*TPase, suggesting that T1 progeny carrying the HPT selection marker, *Ac*TPase, and other T-DNA sequences can be eliminated efficiently solely by GFP and mCherry fluorescence assays.

In a parallel experiment, we assayed 35 pBDL22-transformed T1 rice lines by detecting GFP fluorescence and PCR amplification of *Ds*/GOI, GFP, HPT, and *Ac*TPase sequences. Marker-free *Ds*/GOI plants were obtained from six of these lines (Table 3). However, two of the pBDL22-transformed T1 lines segregated non-fluorescent plants that showed PCR amplification of T-DNA-based GFP, HPT, and *Ac*TPase, suggesting that a single fluorescent protein marker (i.e., GFP in pBDL22) was less reliable than two fluorescent protein markers (i.e., GFP and mCherry in pBDL23) for counterselection against T-DNA-carrying progeny. Given that non-fluorescent plants derived from the single fluorescent protein-expressing *Ac/Ds* transposon vector pBDL22 might also carry T-DNA sequences, the screening of marker-free transgenic plants depends on both GFP fluorescence assays and additional PCR assays of T-DNA sequences.

The efficiencies of screening marker-free T1 plants from pBDL23 and pBDL22 rice lines were 31.0% (13/42) and 17.1% (6/35), respectively. In terms of the screening step of fluorescence assays, the occurrence of false marker-free transgenic plants was 0 for pBDL23 T1 lines and 25% (2/8) for pBDL22 lines.

### Molecular Characterization of the Transgenes in Marker-Free Rice Lines

**FIGURE 4:**
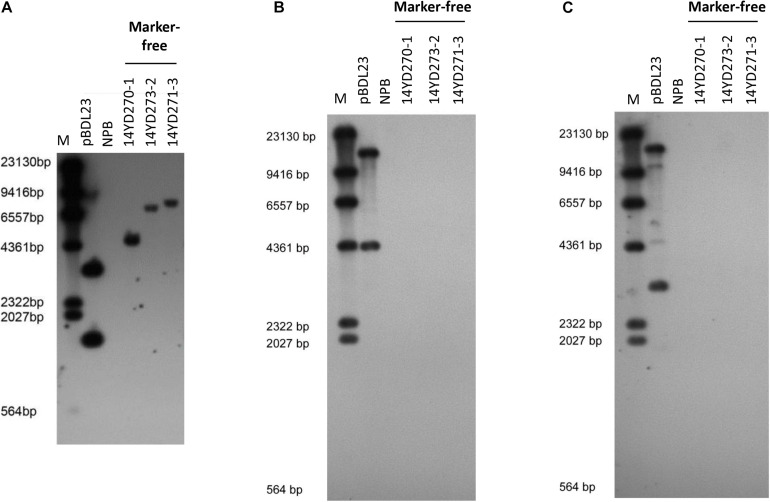
Southern analysis of pBDL23-transformed marker-free T2 plants. Wild-type rice (NPB) was used as a control. **(A)** Southern hybridization using the *Ds* probe. **(B)** Southern hybridization using the *HPT* probe. **(C)** Southern hybridization using the *GFP* probe.

To verify that the marker-free transgenic rice plants transformed with the double fluorescent protein-expressing *Ac/Ds* transposon vector pBDL23 contained *Ds* and the GOI (i.e., the RNAi *Pi21* cassette) but not T-DNA sequences, rice genomic DNAs of marker-free T1 plants from three pBDL23-transformed rice lines were subjected to Southern blot hybridization using *Ds*, GFP, and HPT probes (Figure 4). Marker-free plants from the three rice lines showed distinctive patterns indicative of *Ds* hybridization (Figure 4A), suggesting that the *Ds*/GOI transgene was integrated into different sites in the rice genome. There were no hybridization signals for tested transgenic rice plants using GFP and HPT probes (Figures 4B,C). Therefore, these marker-free transgenic rice plants carried single *Ds* elements but -no T-DNA sequences, confirming that the transformation selection markers were eliminated.

**FIGURE 5:**
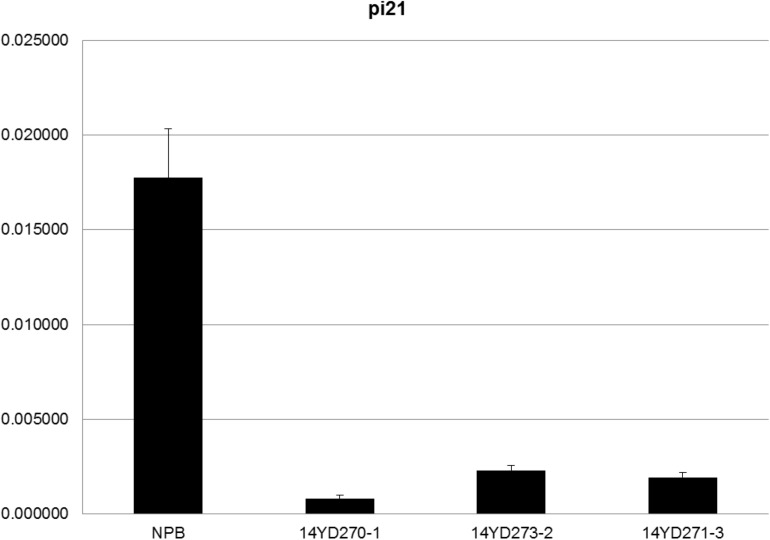
qRT-PCR analysis of *Pi21* transcript levels in pBDL23-transformed marker-free rice. Horizontal axis: Wild-type control (NPB) plants and T2 homozygote plants from transgenic lines 14YD270-1, 14YD273-2, and 14YD271-3, respectively; Longitudinal axis: Relative expression levels of *Pi21* (*OsEF-Tu* was used as an internal reference).

To confirm whether the GOI functions properly in marker-free rice plants, T2 homozygous plants of the three rice lines were analyzed by qRT-PCR to determine the expression levels of the *Pi21* gene (Figure 5). *Pi21* expression levels were significantly suppressed in the marker-free rice plants compared with untransformed Nipponbare (NPB) rice.

**FIGURE 6:**
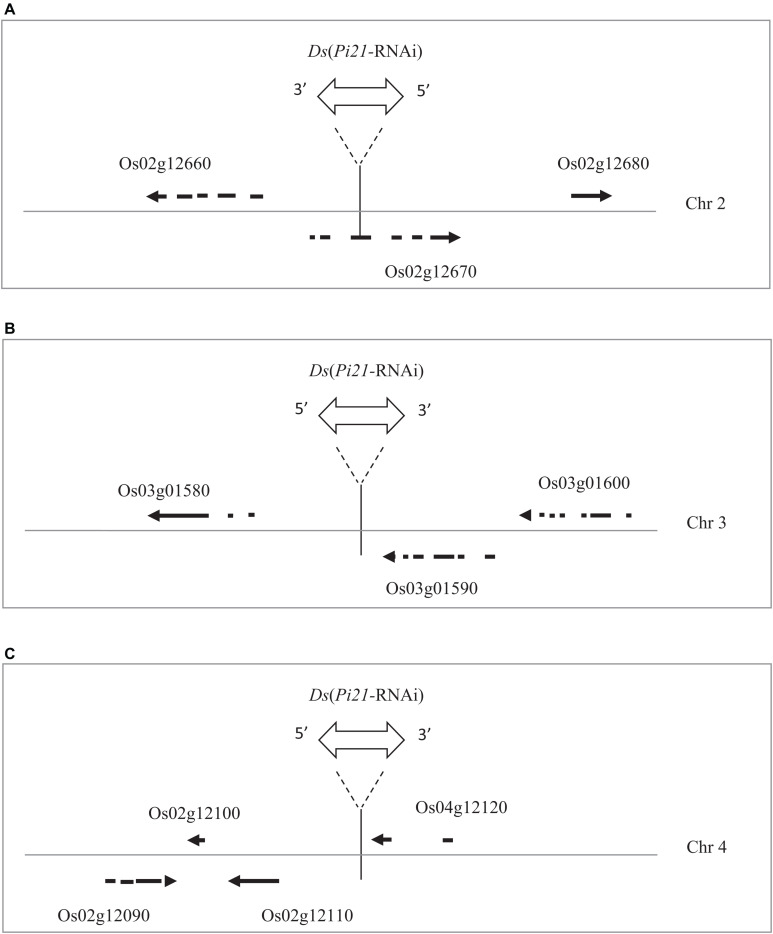
*Ds*(*Pi21-*RNAi) reintegration sites in three marker-free transgenic rice. **(A)** The *Ds* insertion (Chr02:6618501) in the 14YD270-1 rice line disrupted the coding region (CDS) of rice gene Os02g12670. **(B)** The *Ds* insertion (Chr03:369962) in the 14YD273-2 line is 3579 bp upstream from the Os03g01580 CDS and 494 bp downstream of the Os03g01590 CDS. **(C)** The *Ds* insertion (Chr04:6664522) in the 14YD271-3 line is 2864 bp upstream from the Os04g12110 CDS and 205 bp downstream of the Os04g12120 CDS.

Rice sequences flanking *Ds* insertions in marker-free transgenic rice lines 14YD270-1, 14YD273-2, and 14YD271-3 were isolated by thermal asymmetric interlaced (TAIL)-PCR (Supplementary Image 2A). The TAIL-PCR bands were sequenced, and the *Ds* flanking sequences were confirmed by PCR using a *Ds*-specific primer and a primer specific to the rice sequence derived from the TAIL-PCR bands (Supplementary Image 2B). BLAST search in the rice genome was performed using *Ds*-flanking rice sequences of the rice lines. As shown in Figure 6, the reintegrated *Ds* elements in 14YD270-1, 14YD273-2, and 14YD271-3 were mapped into the genomic positions Chr02:6618501, Chr03:369962, and Chr04:6664522, respectively. The *Ds* insertion sites in 14YD273-2 and 14YD271-3 were located in intergenic regions, while the *Ds* in 14YD270-1 was inserted in the coding region (CDS) of rice gene LOC_Os02g12670. Thus, the marker-free transgenic rice lines 14YD273-2 and 14YD271-3 are more suitable for utilization in rice disease resistance breeding.

### Phenotypic Characterization of Marker-Free Transgenic Rice

**FIGURE 7:**
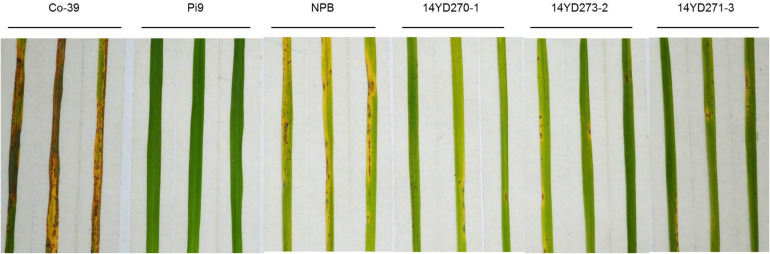
Disease phenotypes of three pBDL23-transformed marker-free rice lines inoculated with rice blast isolate TMC-1. T2 homozygote plants from transgenic lines 14YD270-1, 14YD273-2, and 14YD271-3 were tested; NPB: the transgenic recipient rice line Nipponbare; CO39: a rice blast susceptible cultivar; Pi9: a resistant rice line carrying the *Pi9* rice blast resistance gene (Qu et al., 2006).

To confirm whether the marker-free transgenic plants express rice blast resistance, three pBDL23-transformed marker-free T2 lines (14YD270-1, 14YD273-2, and 14YD271-3) were tested in rice blast inoculation. The rice blast lesions and disease scores of the marker-free rice lines (Supplementary Table 2) were significantly smaller than those of NPB rice (Figure 7).

We investigated the agronomic traits of marker-free transgenic rice. For each plant of the 14YD270-1 and 14YD273-2 T2 lines segregated for *Ds*, PCR for *Ds* was performed, and *Ds*-positive and -negative plants of each line were grouped separately. The agronomic traits spike number, flag leaf length, plant height, and spike length were evaluated for each plant. Student’s *t*-tests were conducted for *Ds*-positive and -negative plant groups in the lines 14YD270-1 and 14YD273-2. No significant difference was found between the *Ds*-positive and -negative groups in the same line for the above-mentioned agronomic traits (Table 4 and Supplementary Image 3). In addition, the heading date of each row of *Ds*-positive and -negative plants was investigated. The average heading dates of the *Ds*-positive and -negative groups of the 14YD270-1 line were 147.75 days, and the average heading dates for the *Ds*-positive and -negative groups in the line 14YD273-2 differed by only 1 day. Therefore, *Ds* insertion did not affect spike number, flag leaf length, plant height, spike length, and heading date in the 14YD270-1 and 14YD273-2 marker-free transgenic rice lines.

**TABLE 4:** Evaluation of agronomic traits of two pBDL23-transformed marker-free rice lines.

	Spike number	Flag leaf length (cm)	Plant height (cm)	Spike length (cm)	Heading date (day)
14YD270-1(*Ds*+)	11 ± 5	29.50 ± 4.57	72.71 ± 7.96	15.89 ± 1.99	147.75
14YD270-1(*Ds−*)	12 ± 4	29.21 ± 3.87	72.37 ± 6.73	15.63 ± 1.69	147.75
*t*-test	*−*0.242	0.167	0.116	0.348	ND
*P*-value	0.81	0.87	0.91	0.73	ND
14YD273-2(*Ds*+)	13 ± 4	21.97 ± 2.14	59.76 ± 4.70	14.32 ± 1.01	147.25
14YD273-2(*Ds−*)	13 ± 3	23.08 ± 2.09	62.08 ± 3.72	14.88 ± 1.65	148.25
*t*-test	0.523E-01	*−*1.22	*−*1.26	*−*0.982	ND
*P*-value	0.96	0.24	0.22	0.34	ND

*For *t*-tests, *Ds*-negative (*Ds−*) plants in the marker-free transgenic lines were used as control in comparison with the *Ds*-positive (*Ds*+) plants. ND, not determined.*

## Discussion

We generated marker-free transgenic rice resistant to rice blast disease by using a double fluorescent protein-expressing *Ac/Ds* vector. The vector for rice transformation harbors mCherry and GFP markers, which facilitated the genetic screening of segregating transgenic populations.

*pi21* is a recessive gene conferring partial resistance to rice blast (Fukuoka et al., 2009). Japanese upland rice cultivars carrying *pi21* recessive loci continue to maintain resistance to rice blast after one century of field cultivation (Fukuoka and Okuno, 2001). *Pi21*-RNAi transgenic rice shows resistance to *M. oryzae* (Fukuoka et al., 2009; Zhang et al., 2016). Rice *pi21* mutants obtained by CRISPR/Cas9 are also resistant to *M. oryzae* (Yang et al., 2017; Li S. F. et al., 2019). Rice carrying a recessive *pi21* gene has limitations when applied to hybrid rice, because both parents of a hybrid need to be backcrossed to a *pi21* donor line to inherit a recessive locus or the *Pi21* genes in both parents need to be knocked out. In this study, the *Pi21*-RNAi expression cassette in marker-free transgenic rice was genetically dominant, and only one of the parents in hybrid rice must carry the *Pi21*-RNAi cassette.

Krattinger et al. (2016) transformed the wheat rust disease resistance gene *Lr34* into rice and developed transgenic rice lines resistant to rice blast. However, the wheat-derived *Lr34* disease resistance gene in the rice genetic background causes senescence in leaves and leaf tip necrosis due to gene pleiotropism. The recessive locus of *pi21* in natural rice populations has undergone natural selection over a long period. Therefore, transgenic modification of the *Pi21* gene is strongly suitable for the genetic improvement of rice blast resistance.

Cao et al. (2020) used a co-transformation method to transform an RNAi expression cassette of a lipoxygenase gene into wheat and analyzed the T-DNA flanking sequences in two marker-free transgenic lines. LB and RB regions of the T-DNA insertion site in one marker-free wheat line had 14 and 21 bp deletions, respectively. A 19 bp deletion and 12 bp insertion also occurred in the wheat genomic region flanking the RB. In the other marker-free line, the LB and RB regions also had 14 and 21 bp deletions, respectively, and a 40 bp deletion occurred in the wheat genomic sequence flanking the RB. InDel events in T-DNA borders and the adjacent plant genomic sequences may potentially affect the stability of GOI expression and the near-neighboring plant genes. Pan et al. (2020) developed marker-free transgenic rice by co-transforming the rice blast resistance gene *Pi9* and an HPT marker, and sequence deletions were located in the T-DNA borders on the basis of the results of PCR analysis of T-DNA boundary sequences. Given that T-DNA transformation is usually accompanied with base deletions, insertions, and complex structure in T-DNA integration sites (Li et al., 2012; Zhao et al., 2013; Gelvin, 2017), the co-transformation and site-specific recombination methods that are based on *Agrobacterium*-mediated T-DNA transformation are prone to the occurrence of complex T-DNA structures and transgene sequence alterations, which can affect the expression of GOI genes. Transgenic rice derived from *Ac/Ds* transposon-mediated transgene reintegration has clear transgene boundary sequences between the *Ds* and the recipient genomic sequence, thereby facilitating stable expression of the transgene in rice.

The single GFP-expressing *Ac/Ds* vector with a *Bacillus thuringiensis* (*Bt*) gene loaded in *Ds* transposable element (Gao et al., 2015) was used to develop marker-free insect-resistant transgenic rice. However, as a result of *Ds* reinsertion inside the GFP gene or a defective GFP gene from T-DNA sequence rearrangement during plant transformation, some plants still carried T-DNA sequences, including the transformation selection marker, among the non-fluorescent progeny. This phenomenon suggested the unreliability of the single GFP selection system (Gao et al., 2015). According to published data from this study (Gao et al., 2015; Table I), among the 28 T1 rice lines that segregated non-fluorescent plants and whose T-DNA-based HPT and GFP were assayed in PCR, eight lines had T1 progeny that were non-fluorescent but still carried T-DNA sequences; the frequency of T1 lines segregating false marker-free plants was 28.6% (8/28). In this study, for the single fluorescent protein vector pBDL22, which was used as a control, 12 T1 rice lines segregated non-fluorescent plants, and two rice lines segregated non-fluorescent plants carrying residual T-DNA sequences, resulting in 16.7% (2/12) of the T1 lines segregating false marker-free transgenic plants. In this study, precise screening of T-DNA-negative plants from segregating populations was achieved by using mCherry and GFP fluorescent protein markers. The double fluorescent protein-expressing *Ac/Ds* vector system is highly suitable for the large-scale screening of transgenic progeny.

The efficiency of generating marker-free transgenic plants via *Ac/Ds*-mediated transgene reintegration can be evaluated by comparing the frequency of marker-free *Ds*/GOI plants in segregating transgenic populations, i.e., the percentage of T1 lines that segregated marker-free *Ds*/GOI plants among all T1 lines (Gao et al., 2015). Using the double fluorescent protein-expressing *Ac/Ds* vector, we efficiently screened segregating transgenic rice populations and obtained marker-free T1 plants from 30.91% (13 among 42) of the rice lines. This finding was comparable with the 26.1% frequency of marker-free *Ds*/GOI plants with the single fluorescent protein-expressing *Ac/Ds* vector (Gao et al., 2015). Although a limited number of pBDL23 transgenic lines were screened, none of the mCherry- and GFP-fluorescence negative plants of the T1 generation showed amplification of T-DNA sequences in the PCR assay. By contrast, the transgenic lines of the control vector pBDL22 and the transgenic T1 lines of Gao et al. (2015) showed that the non-fluorescent phenotypes in multiple lines did not match the PCR assay results. For mCherry and GFP fluorescent protein marker-aided selection, fluorescence assays of the transgenic progeny can be relied on to remove marker genes and T-DNA sequences; the complicated PCR assays of T-DNA sequences and Southern hybridization verification become unnecessary or less dependent on. Therefore, the reliability of mCherry and GFP marker-aided selection was better than that of single fluorescent marker-aided selection of the GFP-expressing *Ac/Ds* vector (Gao et al., 2015).

The *Ds*-positive and -negative plants in two marker-free T2 lines that segregated for *Ds* were grouped for the characterization of agronomic traits of spike number, flag leaf length, plant height, spike length, and heading dates. No significant differences were observed between the *Ds*-positive and -negative plant groups of the same lines, suggesting that *Ds* insertion did not affect the abovementioned agronomic traits. Meanwhile, plant height and flag leaf length of the rice line 14YD273-2 were significantly smaller than those of the 14YD270-1 rice line. These two lines were derived from two independent transformation events, and the differences in some of their agronomic traits might be derived from somaclonal variation. The progenitor T0 plants of the two rice lines may undergo different somatic mutations, resulting in varying genetic backgrounds (Bregitzer et al., 2008). As suggested by Bregitzer et al. (2008), the marker-free *Ds* lines must be backcrossed with the recipient rice, in order to remove genetic background differences.

In this study, we used *Ac/Ds* mediated transgene reintegration method to introduce an RNAi expression cassette of the rice *Pi21* gene that negatively regulates rice blast resistance (Fukuoka et al., 2009) into a susceptible rice cultivar, and developed disease-resistant marker-free transgenic rice lines. Given that transposon-mediated transgene reintegration has distinct advantages, including intact transgene insertion and a low demand on the number of primary transgenic plants, the double fluorescent protein-expressing *Ac/Ds* transposon vector introduced here may be useful for the marker-free transgenic improvement of rice disease resistance and other agronomic traits.

## Materials and Methods

### DNA Vector Construction

The “CaMV35S:mCherry:Tnos” expression cassette was amplified from pBDL09 using the following PCR primers: mCherryinF (5′GGGGGCCCGGTACCGAGCTCCCCC TCAGAAGACCAG3′) and mCherryinR (5′ATGATTACGAA TTCGAGCTAGCTAGTAACATAGATGACACCGC3′). The large fragment of *Sac*I-digested pLJ26 (Gao et al., 2015) was ligated with the PCR-amplified “CaMV35S:mCherry:Tnos” cassette using an In-Fusion HD Cloning kit (Clontech, United States) to construct pBDL10A. One of the PCR primers was designed in such a way that the resulting pBDL10A construct retained a single *Sac*I restriction site between the mCherry and GFP cassettes (likewise, in each of the following In-Fusion Cloning steps using *Asc*I, one primer was designed in a similar way to retain a single *Asc*I site in each new construct). The *Ds* fragment isolated from *Sac*I-digested pLJ26 was inserted into the *Sac*I site in pBDL10A to obtain pBDL11. The rice Actin 1 promoter was amplified from pZJ63 using PCR primers BDL14-F4 (5′CTAATTGAATGGaGCGCCGTCGAGGTCATTCATATGC T3′) and BDL14-R (5′GCAGGAATTCGGCGCCCCTACAAAAA AGCTCCGCAC3′). The Actin 1 promoter was inserted into the *Asc*I site inside the *Ds* of pBDL11 via In-Fusion Cloning to construct pBDL14. The pBDL14 vector retained a single *Asc*I site downstream of the Actin 1 promoter. T3A (the *Pisum sativum* RbcS-3A polyA signal sequence) was amplified from pZJ70 using primers PBDL15-F (5′TTTTTGTAGGGGCGCGCCAGCTTTCGTCCGTATCATC G3′) and PBDL15-R1 (5′GCAGGAATTCGGAGCACCTCGACA AAAAGCCTATAC3′) and then inserted into the *Asc*I site, downstream of the Actin 1 promoter, in pBDL14 by In-Fusion cloning to construct pBDL15. The Pi21 -RNAi cassette for the expression of rice blast resistance (Fukuoka et al., 2009; Zhang et al., 2016) was digested from pBDL18 using *Asc*I and then cloned to a position between the Actin 1 promoter and T3A in pBDL15, thereby constructing the double fluorescent protein-expressing *Ac/Ds* transposon vector pBDL23.

The *Pi21*-RNAi cassette was cloned into the *Ds* element in pLJ26 (Gao et al., 2015) to construct a single fluorescent protein-expressing *Ac/Ds* transposon vector pBDL22. pBDL22 was used as a control for comparison with the double fluorescent protein-expressing vector pBDL23, which was constructed as follows. First, the Actin 1 promoter was amplified from pBDL07 and inserted into the *Asc*I site within the *Ds* of pLJ26 by In-Fusion cloning to construct pBDL20. T3A was then amplified from pBDL07 and inserted into the *Asc*I site downstream of the Actin 1 promoter in pBDL20 by in-fusion cloning to construct pBDL21. The *Pi21*-RNA cassette was released from pBDL18 by *Asc*I digestion and inserted between the Actin 1 promoter and T3A in pBDL21 to obtain pBDL22.

The full-length sequences of pBDL11, pBDL23, and pBDL22 are deposited in the GenBank sequence database (their GenBank accession numbers were MW653811, MW653812, and MW653813, respectively).

### Rice Transformation and Fluorescence Assays of Transformed Rice Plants

pBDL23 and pBDL22 were transformed into *Agrobacterium* strain EHA105 via electroporation. Rice transformation was performed as previously described (Gao et al., 2015; Chen et al., 1998). The seeds of rice cultivar NPB were dehusked, surface-sterilized, and cultured on NB medium containing 2,4-D to induce embryogenic calli. Rice calli were co-cultivated with *Agrobacterium* strains harboring pBDL23 and pBDL22, respectively, for 3 days. The calli were then washed with sterilized water to remove *Agrobacterium* cells and cultured on NB medium containing 50 mg/L hygromycin. Hygromycin-resistant rice calli derived from one or two rounds of selection culture were transferred onto a differentiation medium to regenerate rice plants. The hygromycin-resistant rice plants were examined under a lamp-type fluorescence detector (Fluorescent Protein Macro Detector Set, Biological Laboratory Equipment Maintenance and Service Ltd., Budapest, Hungary) to detect GFP and mCherry fluorescence signals, which were used as criteria to select T0 plants. For BDL23, double-fluorescent (GFP+ and mCherry+) rice plants were selected while single-fluorescent (GFP+ or mCherry+) and non-fluorescent (GFP- and mCherry-) plants were discarded. For pBDL22, GFP-fluorescent T0 plants were selected. Rice transformants were transplanted to soil, and T1 rice seeds were harvested at the maturity stage.

### Rice Seed Germination and Fluorescence Assays of T1 Transgenic Plants

Independently transformed rice lines with at least 100 T1 seeds per line were tested for transgene segregation analysis and marker-free selection. For NPB and each of the pBDL23 and pBDL22 T1 lines, 100 seeds were soaked in water at 37°C for 2 days. The seeds were then washed with water, wrapped in a wet towel, and covered with plastic wrap to retain moisture; incubation continued at 37°C. After 5 days of germination, rice seedlings were observed under the BLS lamp-type fluorescence detector with blue and green excitation light sources to detect GFP and mCherry fluorescence, respectively, to distinguish between mCherry- and GFP-positive plants. Fluorescent and non-fluorescent plants from each line were counted, and chi-square statistical tests were performed. Fluorescent and non-fluorescent plants from each T1 line were grown together in the same pot and kept in a Conviron PGR15 plant growth chamber (Canada) with a photoperiod of 14 h (light)/10 h (dark), light intensity 4 (370 μmol/m^2^/s), 26°C (light)/24°C (dark), and 85% humidity.

### Rice Genomic DNA Extraction

To extract rice genomic DNA, we grew germinated T1 rice seeds in soil for 8 days, and leaf tissue was then sampled from rice seedlings. To screen marker-free transgenic plants, we sampled an equal amount of leaf tissue from each of five non-fluorescent plants from the same T1 line (i.e., to create a DNA pool), and these samples were mixed for rice DNA extraction. The DNA pools were PCR assayed to detect *Ds* and the GOI (see below). When DNA pools showed positive PCR assays for *Ds* and the GOI, rice DNA was extracted from each of the single plants from each pool and assayed individually by PCR. The steps of rice DNA extraction were as follows: 2 cm of leaf tissue was cut from each seedling and placed into a 1.5 ml centrifuge tube, which was added with steel beads and 500 μl of TPS extraction solution (0.1 M Tris-HCl, 0.01 M EDTA, 1 M KCl, pH 8.0). Rice leaf tissue was homogenized using a Tissuelyser-FE III tissue grinder (Jingxin Industrial Development, Shanghai, China) at 70 Hz for 90 s. Homogenized leaf tissue was incubated in TPS extraction solution at 75°C for 30 min (in a water bath) and centrifuged at 12,000 rpm (Centrifuge5430, Eppendorf, Germany) for 10 min. About 200 μl of supernatant was transferred to a new tube and mixed with an equal volume of isopropanol. This mixture was placed at −20°C for 30 min and centrifuged at 12,000 rpm (Centrifuge5430, Eppendorf, Germany) for 5 min. The DNA pellet was briefly washed with 75% of ethanol, air-dried, and dissolved in 50 μl of double-distilled H_2_O containing 0.2 mg/ml RNase A.

### PCR Amplification of *Ds* Empty Donor Sites

The *Ds* transposon empty donor sites (EDS) in pBDL23-transformed rice plants were amplified using the PCR primers GFP-EF (5′ACAACCACTACCTGAGCACC3′) and mCherry-ER (5′TTGGAGCCGTACATGAACTG3′), and the primers GFP-EF and HPT-ER (5′ATCGAAGCTGAAAGCACGAG3′) were for the EDS-PCR of pBDL22 transformants. The cycling parameters for EDS-PCR were as follows: 95°C for 5 min; 33 cycles of 95°C for 30 s, 58°C for 30 s, and 72°C for 2 min; and 72°C for 7 min. The primer pair of mCherry-F1 (5′CAAGGCCTACGTGAAGCACC3′) and TNOS-R (5′CCATCTCATAAATAACGTCATG3′) and the pair of GFP-EF and TNOS-R were used to amplify mCherry and GFP, respectively. The cycling parameters for mCherry and GFP were as follows: 95°C for 5 min; 33 cycles of 95°C for 30 s, 58°C for 30 s, and 72°C for 1 min; and 72°C for 7 min.

### PCR Assays of Transgene Sequences in Rice DNA

PCR primers specific to pBDL22 and pBDL23 T-DNA sequences are listed in Supplementary Table 1. Rice DNA pools derived from the non-fluorescent T1 segregants were PCR assayed using four pairs of primers specific to *Ds* and the GOI. DNA pools showing positive PCR assays for *Ds* and the GOI were further analyzed using primers specific to T-DNA sequences other than *Ds* and the GOI. For DNA pools showing negative results for PCR assays of T-DNA sequences, rice DNA was extracted from individual plants corresponding to each pool and assayed by PCR for both *Ds* and the GOI to screen for marker-free *Ds*/GOI plants. PCR reactions were performed on an ABI Applied Biosystems Veriti 96-Well Thermal cycler (Gene Company, United States) with *Taq* DNA polymerase (TsingKe Biological Technology, China). The cycling parameters for both GOI and *Ds* assays were as follows: 95°C for 5 min; 36 cycles of 95°C for 30 s, 56°C for 30 s, and 72°C for 1 min; and 72°C for 7 min. The cycling parameters for T-DNA assays were as follows: 95°C for 5 min; 26 cycles of 95°C for 30 s, 60°C for 30 s, and 72°C for 1 min; and 72°C for 7 min.

### Southern Blot Hybridization

Southern hybridization was carried out using a Southern blot hybridization kit (Towin Biotechnology, China). About 10 μg of rice DNA was first digested with *Eco*RI (for the *Ds* probe), *Hin*dIII (for the HPT probe), or *Bam*HI (for the GFP probe). The digestion was then loaded onto an agarose gel and electrophoresed at 30V for 16 h. Enzymatic products fractionated in the electrophoresis gels were transferred onto nylon membranes and fixed by UV light. About 30 min of pre-hybridization at 37°C was followed by overnight hybridization with a digoxigenin-labeled probe. The hybridization signal was revealed by reaction with the chemiluminescent substrate CSPD and detected visually by X-ray. We used 822 bp, 721 bp, and 840 bp PCR fragment sequences as hybridization probes for the detection of *Ds*, GFP, and HPT, respectively, as described previously (Gao et al., 2015).

### Rice RNA Extraction

In this study, 2 cm of rice leaf tissue was sampled from single rice seedlings and placed into a 2 ml centrifuge tube. Steel beads were added, and the tissue samples were homogenized at 50 Hz for 90 s using a tissue grinder after liquid nitrogen precooling of centrifuge tube racks. Subsequently, 1 ml of Trizol was added to the leaf powder, and the mixture was incubated at room temperature for 5 min. Rice RNA was extracted by adding 200 μl of chloroform/isoproterenol (24:1) and centrifuging the resulting solution at 12,000 rpm (Centrifuge5415R, Eppendorf, Germany) for 10 min at 4°C. The supernatant was transferred to a new tube to which 500 μl of isopropanol was added. The solution was gently mixed and stored at −20°C for 10 min and then centrifuged at 12,000 rpm (Centrifuge5415R, Eppendorf, Germany) and 4°C for 10 min. The nucleic acid precipitate was washed by adding 1 ml of 75% ethanol, followed by centrifugation at 9,900 rpm (Centrifuge5415R, Eppendorf, Germany) and 4°C for 5 min and drying in air. The resulting RNA pellet was then dissolved in RNase-free water and stored at −80°C.

### qRT-PCR

Reverse transcription of rice RNA was performed using the TransScript All-in-one First Strand cDNA Synthesis SuperMix for qPCR (TransGen Biotech, China) Reverse Transcription Kit as per the manufacturer’s instructions. qPCR was carried out with the TranStart Top/Tip Green qPCR SuperMix (TransGen Biotech, China) kit using the Bio-Rad CFX96 Real-time System. The cycling parameters were as follows: 94°C for 30 s; 39 cycles of 94°C for 5 s, 58°C for 15 s, and 72°C for 10 s. *OsEF-Tu* was used as an internal reference gene, and relative expression levels of the target gene were calculated by the 2^–Δ^ ^Δ^ CT method using three technical replicates for each sample. The *OsEF-Tu* primers were EF-Tu-F (5′ATGATCACGGGTACCTCCCA3′) and EF-Tu- R (5′TTGTCAGGGTTGTAGCCGAC3′); the *Pi21* primers were Pi21-RT-F (5′CGGCAAATTTGACAGATGGGTAT3′) and Pi21-RT-R (5′CTTCTCCGGGTCGAACTTC3′).

### TAIL-PCR of *Ds* Flanking Sequences

*Ds* flanking sequences in marker-free transgenic rice lines were amplified by TAIL-PCR (Liu et al., 1995) and hiTAIL-PCR (Liu and Chen, 2007). Two to three successive rounds of nested PCR reactions were performed using the primers specific to the 5′ *Ds*- and 3′ *Ds* terminal sequences (Kolesnik et al., 2004) and arbitrary degenerate (AD) primers. For each TAIL-PCR or hiTAIL-PCR reaction, a 5′ *Ds*- or 3′ *Ds*-specific primer was paired with an AD primer (AD2; Liu et al., 1995) or a long arbitrary degenerate (LAD) primer (LAD-1, LAD-2, LAD-3 or AC1; Liu and Chen, 2007). PCR products were fractionated on 1 to 1.5% (w/v) agarose gels. PCR bands were cut from the gels and purified for sequencing. The *Ds* flanking sequences were confirmed by PCR using a *Ds*-specific primer and a primer specific to the rice sequence derived from the sequencing results of TAIL-PCR bands.

### Rice Blast Disease Inoculation

Rice blast disease inoculation was performed as previously described (Yang et al., 2017) with slight modifications. Rice blast spores that had been stored on filter paper at −20°C were cultured on CM medium at 25°C with a photoperiod of 12 h light/12 h dark. After culturing for 12 days, rice blast spores were washed from CM medium using sterile water containing 0.02% Tween 20. The collected spores were counted under a microscope using a hemocyte counting plate. Spore concentration was adjusted to 5 × 10^5^ spores/ml with 0.02% Tween 20 (Li et al., 2016). Germinated rice seeds were sown in soil pots and grown for 10 days in a Conviron plant growth chamber (Canada) with a photoperiod of 14 h (light)/10 h (dark), light intensity 4 (370 μmol/m^2^/s), and 26°C (light)/24°C (dark) at 85% humidity. Each pot contained 18–20 rice seedlings, and 3.5 ml of the spore suspension was used for rice blast inoculation. About 2 ml of spore suspension was initially sprayed onto the rice seedlings using a spray gun to coat all leaves with water droplets. Rice seedlings were then protected using a plastic bottle whose cap and bottom had been removed, and 1.5 ml of spore suspension was sprayed into the mouth of the bottle to ensure that the bottle remained full of fog. The bottle mouth was then sealed with plastic wrap. The rice seedlings were kept in a plant growth chamber at 25°C in the dark for 34 h, after which the photoperiod of 26°C (light)/24°C (dark) was resumed. Disease symptoms were examined 5 days post inoculation.

### Evaluation of Agronomic Traits of Marker-Free Transgenic Rice

Rice seeds of marker-free T2 transgenic rice lines that segregated for *Ds* (GOI) were planted. Total genomic DNA was extracted from 50 mg of leaf tissue sampled from each rice seedling for PCR assay of *Ds*. *Ds*-positive and -negative T2 plants were grouped separately. For the *Ds*-positive and -negative plant groups of each transgenic rice line, each group was planted into two rows. Spike number, flag leaf length, plant height, and spike length were investigated for each plant. Spike number was counted at rice maturity. Five tillers were obtained randomly for each plant to measure the flag leaf length and spike length separately, and average flag leaf length and average spike length of individual plants were calculated. Date of rice heading was observed for each row (six plants in total) of *Ds*-positive or negative group, and the date of 50% plants heading was taken as the date of rice heading. The number of days from the date of root and bud emergence during seed germination to the date of rice heading was calculated as the heading date. The average heading date of two rows of *Ds*-positive or negative plants was calculated for each T2 transgenic rice line.

## Data Availability Statement

The original contributions presented in the study are publicly available. This data can be found here: https://www.ncbi.nlm.nih.gov/, Genbank accession numbers: MW653811, MW653812, and MW653813.

## Author Contributions

SQ designed and supervised the research work. XL, LP, and DB completed the major experiments. XL and SQ wrote the manuscript. All authors made substantial direct and intellectual contribution to the work and approved it for publication.

## Conflict of Interest

The authors declare that the research was conducted in the absence of any commercial or financial relationships that could be construed as a potential conflict of interest.

## Supplementary Material

Supplementary Image 1 PCR detection of the *Ds* (*Pi21*-RNAi) and T-DNA sequences in pBDL23- and pBDL22-transformed T1 plants.

Supplementary Image 2 TAIL-PCR of rice genomic sequences flanking the Ds insertions in marker-free transgenic rice lines 14YD270-1, 14YD273-2, and 14YD271-3.

Supplementary Image 3 Evaluation of rice agronomic traits of two pBDL23-transformed marker-free rice lines.

Supplementary Table 1 PCR primers used to screen marker-free *Ds* plants.

Supplementary Table 2 Disease indexes of three pBDL23-transformed marker-free rice lines inoculated with rice blast isolate TMC-1.
