# Supplementary material for: Generation of Marker-Free Transgenic Rice Resistant to Rice Blast Disease Using Ac/Ds Transposon-Mediated Transgene Reintegration System
Source: Front Plant Sci. 2021 Apr 20;12:644437. doi: 10.3389/fpls.2021.644437 (PMC8095379; doi:10.3389/fpls.2021.644437)
Supplement: Supplementary Image 1 — PCR detection of the Ds (Pi21-RNAi) and T-DNA sequences in pBDL23- and pBDL22-transformed T1 plants. [file Image_1.pdf]

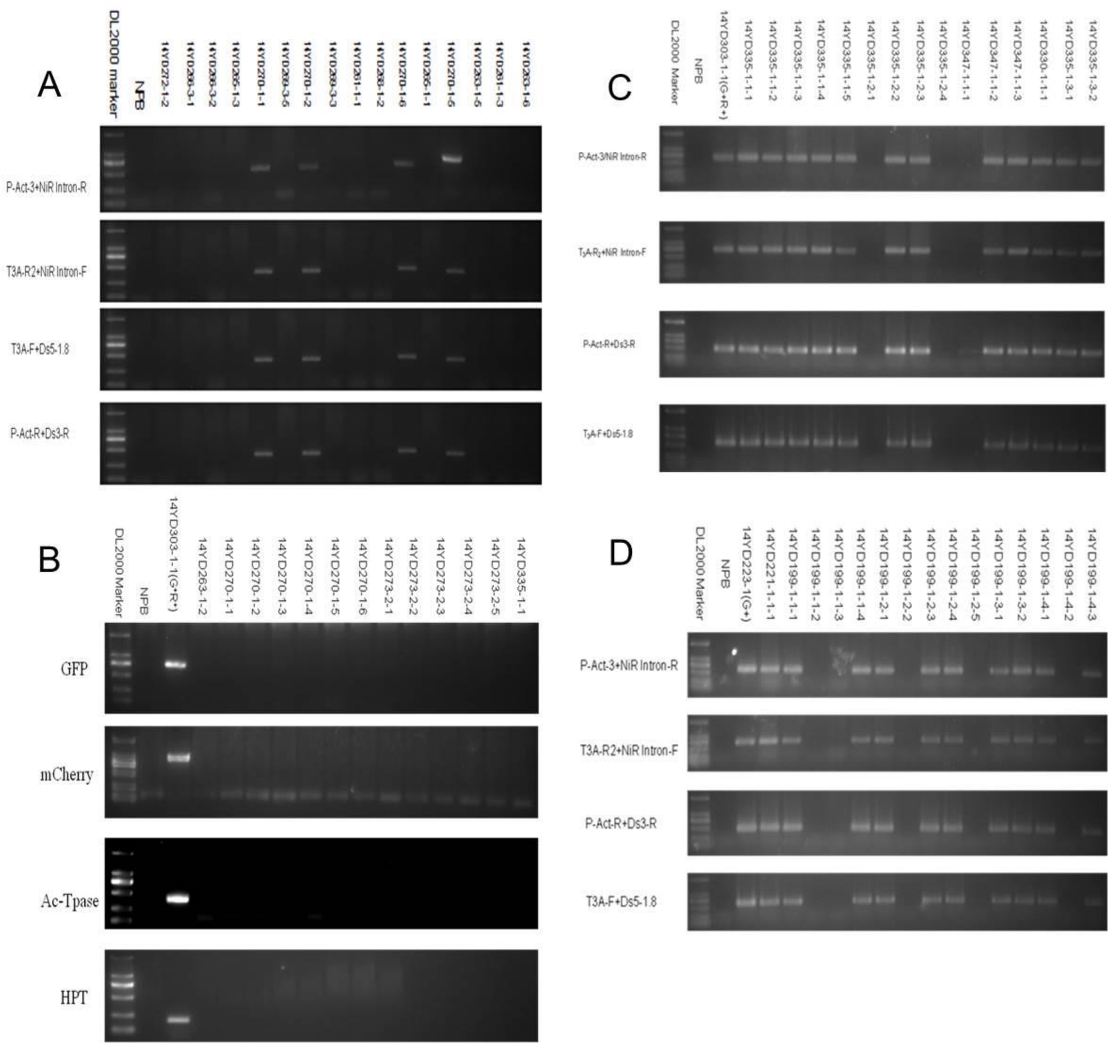

Supplementary Image 1. PCR detection of the *Ds* (*Pi21*-RNAi) and T-DNA sequences in pBDL23- and pBDL22-transformed T1 plants. Nipponbare (NPB) was used as a control. (A) The GOI *Ds* (*Pi21*-RNAi) in the rice DNA pools of pBDL23-transformed nonfluorescent T1 siblings was detected in PCR using four primer pairs. (B) PCR detection of T-DNA sequences in the rice DNA pools described above. (C) PCR detection of the GOI *Ds* (*Pi21*-RNAi) in pBDL23-transformed nonfluorescent T1 individual plants. (D) PCR detection of the GOI in pBDL22-transformed nonfluorescent T1 individual plants.
