# Supplementary material for: Generation of Marker-Free Transgenic Rice Resistant to Rice Blast Disease Using Ac/Ds Transposon-Mediated Transgene Reintegration System
Source: Front Plant Sci. 2021 Apr 20;12:644437. doi: 10.3389/fpls.2021.644437 (PMC8095379; doi:10.3389/fpls.2021.644437)
Supplement: Supplementary Image 2 — TAIL-PCR of rice genomic sequences flanking the Ds insertions in marker-free transgenic rice lines 14YD270-1, 14YD273-2, and 14YD271-3. [file Image_2.pdf]

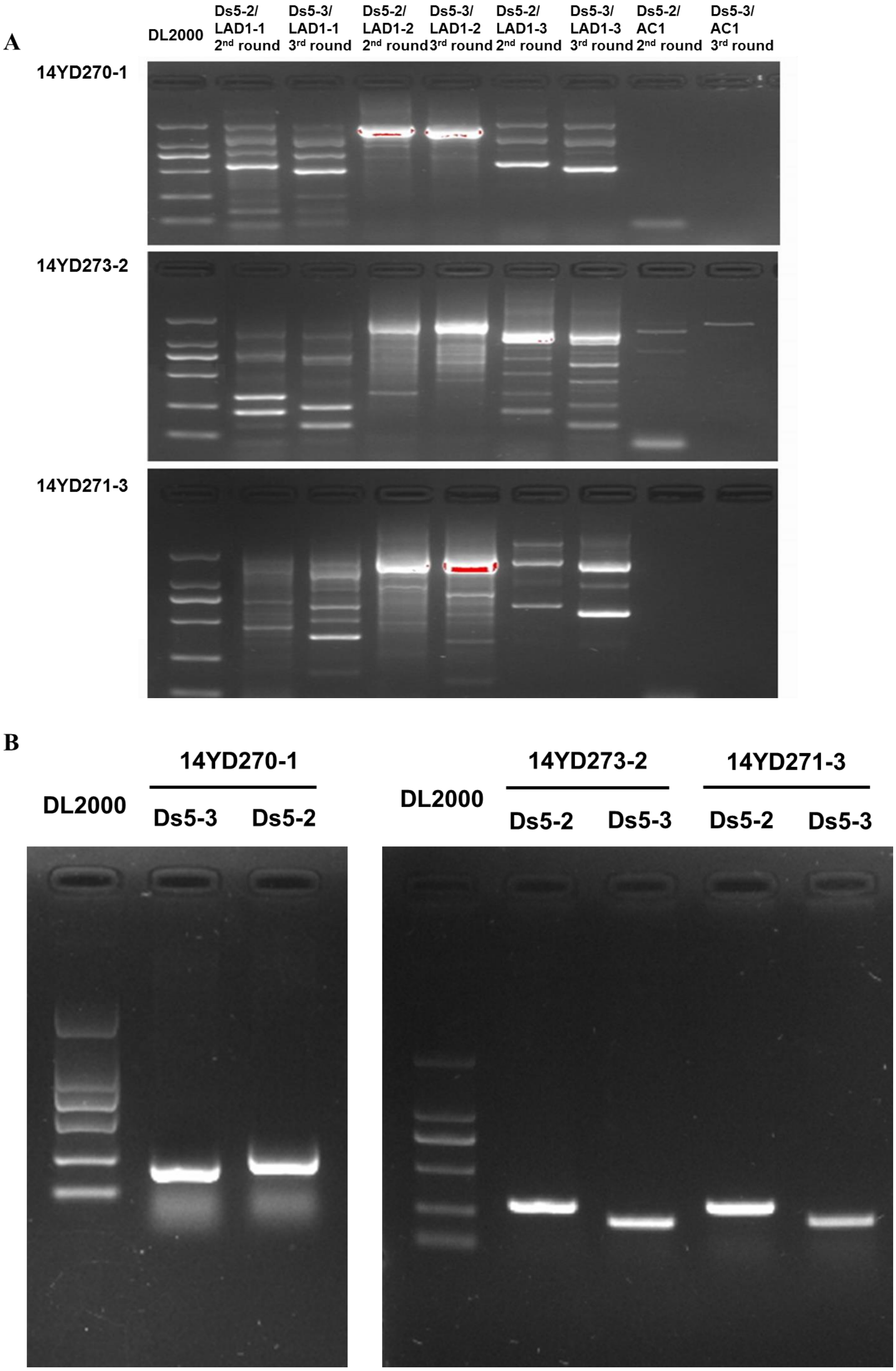

Supplementary Image 2. TAIL-PCR of rice genomic sequences flanking the *Ds* insertions in marker-free transgenic rice lines 14YD270-1, 14YD273-2 and 14YD271-3. DL2000, DNA markers 2000, 1000, 750, 500, 250, and 100 bp (TsingKe Biological Technology, China). (A) TAIL-PCR of the marker-free rice lines was

performed using a primer specific to the *Ds* 5' terminal sequence (Ds 5-2 or Ds 5-3) and arbitrary degenerate (AD) primers (Liu et al., 1995) or long arbitrary degenerate (LAD) primers (Liu and Chen, 2007). (B) *Ds* flanking sequences were confirmed by PCR using a *Ds*-specific primer (Ds5-2 or Ds5-3) and a primer specific to the rice sequence derived from the sequencing results of TAIL-PCR bands.
