# Supplementary material for: Generation of Marker-Free Transgenic Rice Resistant to Rice Blast Disease Using Ac/Ds Transposon-Mediated Transgene Reintegration System
Source: Front Plant Sci. 2021 Apr 20;12:644437. doi: 10.3389/fpls.2021.644437 (PMC8095379; doi:10.3389/fpls.2021.644437)
Supplement: Supplementary Image 3 — Evaluation of rice agronomic traits of two pBDL23-transformed marker-free rice lines. [file Image_3.pdf]

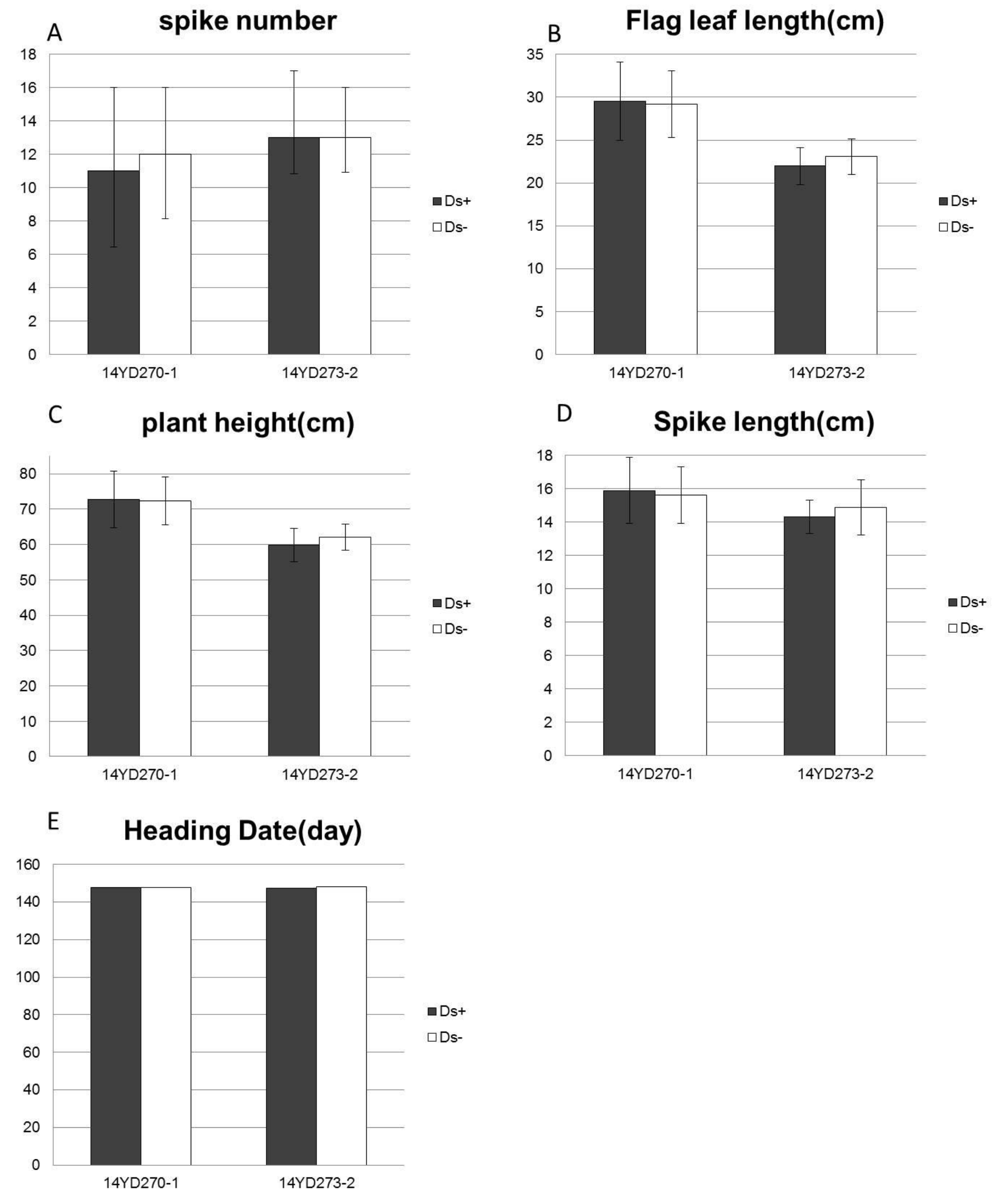

Supplementary Image 3. Evaluation of rice agronomic traits of two pBDL23-transformed marker-free rice lines. *Ds*-negative plants of the marker-free transgenic lines 14YD270-1 and 14YD273-2 were used as control. The p-value in T-test for spike number, flag leaf length, plant height and Spike length is greater than 0.01, indicating no significant difference between the *Ds*-positive and -negative plant groups. (A) spike number. (B) flag leaf length. (C) plant height. (D) Spike length. (E) Heading Date.
