# Supplementary material for: Generation of Marker-Free Transgenic Rice Resistant to Rice Blast Disease Using Ac/Ds Transposon-Mediated Transgene Reintegration System
Source: Front Plant Sci. 2021 Apr 20;12:644437. doi: 10.3389/fpls.2021.644437 (PMC8095379; doi:10.3389/fpls.2021.644437)
Supplement: Supplementary Table 1 — PCR primers used to screen marker-free Ds plants. [file Table_1.pdf]

Supplementary Table 1. PCR primers used to screen marker-free *Ds* plants

Primer pair	Target sequence	Primers sequence（5′ to 3′）
P-Act-3	GOI	GGTAGAATTTGAATCCCTCAGCA
NiR Intron-R		CTGCACAATTTCAAAGATTGAACC
T3A-R2	GOI	AAGAACACAGTAAATTACAAGCAG
NiR Intron-F		CGAGGTAAGTATGCACTTAAAG
P-Act-R	GOI	CTTATACAAATCCCTCTGTATTTAC
Ds3-R		CGGTAATCGAAAACCGATACGAT
T3A-F	GOI	GTTCGTCAAGTTCAATGCATCAG
Ds5-1.8		ACTTGAGAACATCACATAGTTTAGT
35S-mCherry-F	T-DNA	TACAATTACAGGATCCATGGTGAGCA
PlacZ-R		ATTAGGCACCCCAGGCTTTACACTTT
Ac-Tpase-F	T-DNA	GGTTCCCGTCCGATTTCGAC
Ubi-F		ATGGCATATGCAGCAGCTATATG
Ubi-R	T-DNA	CACTGATATTATTGTAGTACTATAG
GFP2-		GGTCACGAACTCCAGCAGGAC
Hyg-F	T-DNA	ACGGTGTCGTCCATCACAGTTTGCC
Hyg-R		TTCCGGAAGTGCTTGACATTGGGGA
