# Supplementary material for: Generation of Marker-Free Transgenic Rice Resistant to Rice Blast Disease Using Ac/Ds Transposon-Mediated Transgene Reintegration System
Source: Front Plant Sci. 2021 Apr 20;12:644437. doi: 10.3389/fpls.2021.644437 (PMC8095379; doi:10.3389/fpls.2021.644437)
Supplement: Supplementary Table 2 — Disease indexes of three pBDL23-transformed marker-free rice lines inoculated with rice blast isolate TMC-1. [file Table_2.pdf]

Supplementary Table 2. Disease indexes of three pBDL23-transformed marker-free rice lines inoculated with rice blast isolate TMC-1

	plant	1	2	3	4	5	6	7	8	9	10	11	12	13	14	15	16	17	18	19	Mean*	P-value**
1	14YD270-1	2	4	2	4	3	3	3	4	4	3	5	3	2	5	5	2	3	3	6	3.5±0.3	3.64578E-08
	NPB	7	7	6	5	7	6	3	6	6	5	7	6	6	7	7	6	5	5	7	6.0±0.2	
2	14YD273-2	4	3	3	3	4	5	2	3	3	3	5	2	4	3	3	3	4	4	4	3.4±0.2	1.33992E-10
	NPB	5	6	7	7	4	5	7	7	7	6	6	5	6	5	5	7	7	7	6	6.1±0.2	
3	14YD271-3	5	3	4	3	4	5	3	2	2	3	6	3	4	2	3	4	5	4	4	3.6±0.8	3.37276E-07
	NPB	7	5	5	6	7	7	6	4	5	5	6	7	7	6	5	5	4	7	7	5.8±1.1	

14YD270-1, 14YD273-2 and 14YD271-3 were T2 homozygote plants of marker-free transgenic lines, in which a *Pi21*-RNAi cassette was carried by the *Ds* element. NPB, the untransformed recipient rice cultivar Nipponbare was used as rice blast-susceptible control.

* Rice plants were inoculated with the rice blast (*M. oryzae*) isolate TMC-1 and disease index were evaluated based on the lesion size on leaves and the proportion of lesion area to total leaf area, according to the International Rice Research Institute (IRRI) rice blast 0-9 rank standard. (Chaudhary, R. C. 1996. Standard Evaluation System of Rice. (4th Edition), IRRI, Manila, Philippines, pp. 52)

** The p-value in T-test is less than 0.01, suggesting significant difference between the marker-free *Ds* (*Pi21*-RNAi) rice lines and NPB.
